# Long‐Term Risk of Rheumatoid Arthritis in Individuals With Autism Spectrum Disorder: A Population‐Based Matched Cohort Study

**DOI:** 10.1002/aur.70349

**Published:** 2026-08-28

**Authors:** Evora Hailin Zhu, Stephen Z. Levine, Matilda Morin, Weiyao Yin, Benjamin Christoffersen, Johan Askling, Natalia Rivera, Abraham Reichenberg, Joseph D. Buxbaum, Sven Sandin, Benjamin Hon Kei Yip

**Affiliations:** ^1^ Jockey Club School of Public Health and Primary Care The Chinese University of Hong Kong Hong Kong SAR; ^2^ School of Public Health University of Haifa Haifa Israel; ^3^ Department of Medical Epidemiology and Biostatistics Karolinska Institutet Stockholm Sweden; ^4^ Churney ApS Copenhagen Denmark; ^5^ Clinical Epidemiology Division, Department of Medicine Solna Karolinska Institutet Stockholm Sweden; ^6^ Rheumatology Division, Department of Medicine Solna Karolinska Institutet, Karolinska University Hospital Stockholm Sweden; ^7^ Department of Psychiatry Icahn School of Medicine Mount Sinai New York USA

**Keywords:** autism, autoimmune, comorbidity, population‐based cohort, rheumatoid arthritis

## Abstract

To investigate the long‐term risk of rheumatoid arthritis (RA) among individuals with autism spectrum disorder (ASD), addressing potential immune comorbidity in ASD populations. A population‐based matched cohort was assembled using Swedish registers, including 46,164 individuals diagnosed with ASD between 1987 and 2017, matched to 4,634,895 controls by sex and birth year. Cohort members were followed from age 18 until RA diagnosis, death, emigration, or end of follow‐up, providing up to 30 years of observation into adulthood. Cox proportional hazards models estimated hazard ratios (HR) for RA, with sensitivity analyses adjusting for parental autoimmune, psychiatric history, and socioeconomic status. RA diagnosis rates were similar between individuals with ASD (*n* = 58, 0.13%) and controls (*n* = 5484, 0.12%), with no increased risk: overall HR = 1.06 (95% CI 0.82–1.37), seropositive HR = 1.00 (0.67–1.48), and seronegative HR = 1.12 (0.79–1.57). Complementary analyses adjusting for parental psychiatric history, autoimmune conditions, and socioeconomic factors yielded consistent null findings (HR = 1.34, 95% CI 0.97–1.84). Sensitivity analyses, including accelerated failure time models and alternative RA subtype definitions, confirmed these results. This large‐scale population study does not suggest an increased RA risk in individuals with ASD. Despite suggested immune dysfunction in ASD and shared genetic pathways with autoimmune conditions, we did not observe evidence that ASD is associated with elevated RA risk during adulthood; In conclusion, these findings inform understanding of autoimmune comorbidity patterns in autism and may guide evidence‐based clinical monitoring for autistic adults.

## Summary

Do autistic adults have a higher risk of rheumatoid arthritis (RA)? RA is a chronic autoimmune disease that causes joint pain, swelling, and stiffness and can lead to long‐term disability if untreated. To explore this question, researchers used Swedish national health data; researchers followed nearly 50,000 autistic people and over 4.6 million non‐autistic people for up to 30 years, comparing who developed RA in each group. RA is a severe autoimmune condition that causes joint pain, swelling, and stiffness. Results were reassuring—autistic adults were not more likely to develop RA than non‐autistic adults. Rates were very similar in both groups. These findings suggest RA is not a particular concern linked to autism and can guide practical health monitoring and care planning for autistic adults and their families.

## 1 Introduction

Autism spectrum disorder (ASD) is a neurodevelopmental disorder characterized by social communication deficits, restricted interests, and repetitive behaviors (First 2013). Beyond these features, individuals with ASD face increased risks of premature death (Catalá‐López et al. 2022) and face multiple co‐morbidities, including cancer (Chiang et al. 2015), obesity (Kahathuduwa et al. 2019), and dementia (Vivanti et al. 2021), highlighting the importance of understanding long‐term health outcomes in this population.

Over the past decades, a growing body of literature has highlighted the role of immune dysfunction in ASD. Dysregulation affects both innate and adaptive immune responses, with elevated pro‐inflammatory cytokines, including interleukin (IL)‐1 (Saad et al. 2020; Xie et al. 2017) and tumor necrosis factor (TNF) consistently observed in individuals with ASD compared to controls (Saad et al. 2020; Xie et al. 2017). This immune dysregulation appears to be both a risk factor for ASD development and a contributor to increased risk of immune‐related comorbidities (Meltzer and Van de Water 2017). Notably, individuals with ASD show elevated rates of allergic manifestations and autoimmune diseases, including inflammatory bowel disease (IBD), compared to the general population (Lee et al. 2018).

The relationship between ASD and autoimmune conditions extends beyond clinical observations to shared biological mechanisms. Autoimmune disorders have highly heterogeneous etiologies. We chose to focus on rheumatoid arthritis (RA), a common autoimmune disease and the most prevalent inflammatory joint disease, and one with a well‐established diagnostic pathway and a biologically feasible pathway to increased ASD risk (Yin et al. 2023; Zhu et al. 2025). The choice is supported by extensive epidemiological literature derived from the Swedish national registers, which provides robust case ascertainment and rich covariate data (Eriksson et al. 2013; Neovius et al. 2011). Both ASD and RA—a chronic autoimmune inflammatory disease—have been associated with specific alleles within the human leukocyte antigen (HLA) region, particularly HLA‐DRB1*04 (Kurkó et al. 2013; Torres et al. 2012). Furthermore, maternal RA is associated with increased risk of ASD in offspring (Atladóttir et al. 2009; He et al. 2022; Yin et al. 2023; Zhu et al. 2020), suggesting potential shared pathways between these conditions. However, whether individuals with ASD themselves face increased risk of developing RA remains unexplored, representing a critical gap in our understanding of autoimmune comorbidity patterns in autism (Ly et al. 2022).

RA affects approximately 1% of adults in developed countries (Uthman 2016), with onset typically occurring in adulthood and incidence peaking in later decades of life, though cases can present as early as Age 18 (Symmons et al. 2002). The condition disproportionately affects women (2.5:1 ratio) and can result in severe joint damage, disability (Aletaha and Smolen 2018), and increased mortality (Watson et al. 2003). Critically, the adult‐onset pattern of RA has created a significant methodological challenge for autism‐autoimmune research: most studies examining autoimmune conditions in ASD populations have been limited by insufficient follow‐up duration to capture the typical age range when RA develops (Hughes et al. 2018). Given the established immune dysfunction in ASD and the chronic, disabling nature of RA, determining whether adults with ASD face elevated RA risk has important implications for clinical monitoring, health planning, and understanding of autism‐related comorbidity patterns. The current study addresses this gap by leveraging a uniquely comprehensive dataset to test the hypothesis that individuals diagnosed with ASD are at increased risk of developing RA. Using a large matched national cohort with up to 30 years of follow‐up into adulthood, we provide real‐world evidence on long‐term RA risk in autism populations.

## 2 Methods

### 2.1 Study Design and Population

A population‐based matched cohort design with delayed entry was implemented. Exposed individuals included all Swedish citizens diagnosed with ASD from 1987 until the end of 2017 (Figure 1). To ensure at least 1 year of follow‐up for RA starting from age 18, only individuals born before 1999 were included. Each individual diagnosed with ASD was matched (by sex and year of birth, at the time of the ASD diagnosis of the index case) to 100 individuals without an ASD diagnosis (Breslow et al. 1983). Resampling was implemented to ensure a sufficient number of the unexposed in a few strata (Breslow 1996). Individuals diagnosed with ASD at age 18 or above, and the corresponding matched control, entered the cohort depending on the date of ASD diagnosis of the index case. Because RA cannot be reliably diagnosed before age 18, cohort entry was defined as age 18 or first diagnosis with ASD, whichever came last. Individuals with missing parental age were excluded because parental age was highly correlated with ASD (Sandin et al. 2016) and included as an important adjustment.

**FIGURE 1:**
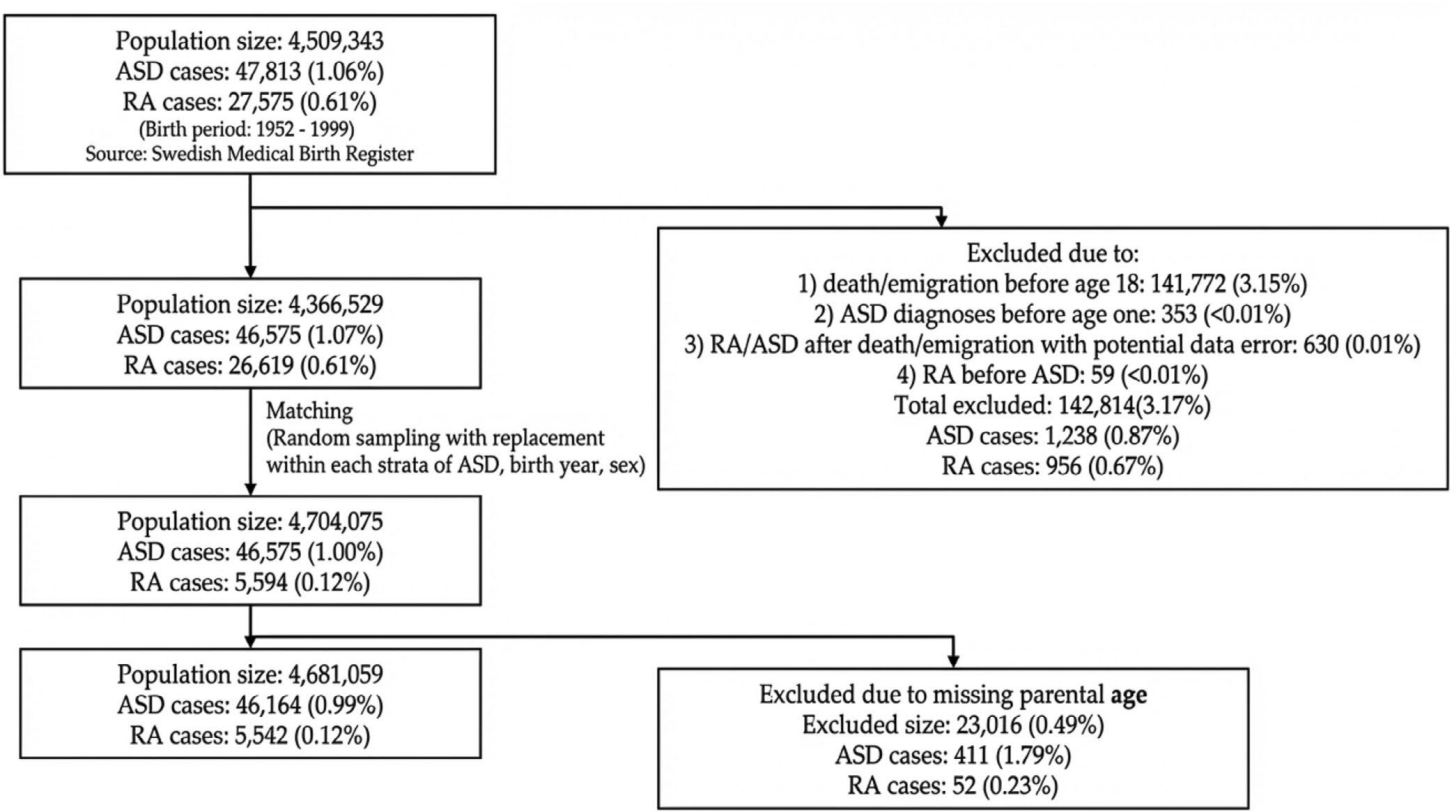
Study flowchart. Abbreviations: ASD, autism spectrum disorder; RA, rheumatoid arthritis.

### 2.2 Setting and Data Sources

The study database was constructed by linking multiple Swedish national registers (Figure S1). The source population was identified in the Swedish Medical Birth Register (MBR), which covers 99% of all birth information nationwide since 1973 (Axelsson 2003). Information to link families was available in the Swedish Multi‐Generation Register, including Swedish citizens born since 1932 (Ekbom 2011). Death and emigration information from the Swedish Total Population Register were both available from 1973 and were used to censor follow‐up time. Diagnostic information was derived from the Swedish National Patient Register (NPR). All inpatient psychiatric diagnoses have been recorded in NPR, nationally, since 1973, and for somatic diseases since 1987 (Ludvigsson et al. 2016). Since 2001 to 2005, incrementally, NPR records outpatient visits to clinical specialists, including day surgery and psychiatric care from both private and public caregivers. The NPR has implemented the International Classification of Diseases 9th revision (ICD‐9) since 1987 and ICD‐10 since 1997 (Quan et al. 2008). This study was approved by the Swedish Ethical Review Agency Stockholm (ref 2017/1875–31/2; 2018/1864–32; 2019–06314). The procedures used to create the analytical dataset from the Swedish national registries are depicted in Figure S1.

### 2.3 Outcome: Rheumatoid Arthritis

Incident RA diagnoses, identified as the first RA diagnosis from age 18 years, from January 01, 1987 to December 31, 2018 were extracted from the NPR. RA diagnoses after birth but before age 18 were excluded. RA subtypes, seropositive, and seronegative, were defined as recorded with different ICD codes (listed and classified in Table S1) in the NPR. The RA diagnoses in the NPR have been used frequently in similar research and have been validated in prior studies (Eriksson et al. 2013; Nørgaard et al. 2010; Waldenlind et al. 2014).

### 2.4 Exposure: Autism Spectrum Disorder

ASD diagnoses were obtained from the NPR. Sweden mandates that all infant and preschool children undergo routine annual medical and developmental evaluations. The developmental evaluation consists of motor skills, language, cognitive, and social development assessments for children at age 4. If a developmental disorder is suspected, a child is referred for further assessment and if confirmed, the diagnosis is recorded in the NPR. Previous validation studies support the reliability of ASD ascertainment in Sweden (Ekholm et al. 2005). ICD version 9 and 10 codes for ASD are presented in Table S1.

### 2.5 Covariates

We extracted information about birth year, offspring sex, and maternal age at delivery from the MBR. Information about fathers and paternal age at delivery was ascertained by linking the MBR with the Swedish Multi‐Generation Register. Maternal RA, maternal, and paternal psychiatric history (yes/no) at childbirth were derived from the NPR. Maternal and paternal maximum attained educational level at delivery (< 9; ≥ 9 years in school) were obtained from the Swedish education register. Maternal and paternal income were from the Longitudinal Database for Health Insurance and Labor Market Studies (LISA) (Ludvigsson et al. 2019), which provides information on individual and household income measures.

### 2.6 Statistical Analysis

We quantified the risk of RA among individuals diagnosed with ASD compared to those without ASD using hazard ratios (HRs) together with their associated two‐sided 95% confidence intervals (CI) from fitting Cox proportional hazards regression models with attained age as the underlying time scale. Cohort entry was defined as age 18 or the first diagnosis with ASD, whichever came last. This definition was chosen because RA cannot be reliably diagnosed before this age, and the follow‐up period between earlier ASD and age 18 was not counted accordingly. Each individual was followed from the time of cohort entry until death, emigration, end of the follow‐up on December 31, 2018, or the diagnosis of RA, whichever occurred first. To address potential correlations in the data due to resampling and the potential for within‐family correlations, robust sandwich standard errors (Hardin 2003) were calculated. To assess the proportional hazards assumption of the fitted Cox model, a visual inspection was conducted of the plotted Schoenfeld residuals versus age (Frome and Checkoway 1985).

As our baseline model, we fitted a (“crude”) model adjusted by birth year and sex, in which birth year was fitted using natural cubic splines (Hastie et al. 2009) with three degrees of freedom (default implementation in SAS procedure PHREG) and sex as a categorical covariate. Next, we extended the crude model by adding parameters for maternal and paternal age, each using natural cubic splines with three degrees of freedom (adjusted model).

For descriptive purposes, we estimated the cumulative incidence of RA in subgroups of ASD exposure by plotting predicted values estimated from Cox regression (Nieto and Coresh 1996). We calculated the age‐adjusted incidence rates of RA (per 100,000 person‐years) and their associated 95% CIs from Poisson regression models. The Poisson regression model incorporated age at follow‐up, sex (male/female), and an offset parameter representing the number of person‐years at risk in each age–sex combination.

### 2.7 Complementary Analyses

We chose the sub cohort as individuals born from 1985 to 1999 to examine potential confounding, which is not feasible in the main analysis. We implemented two complementary analyses of individuals born during later birth years (1985–1999), and we further conducted three additional sensitivity analyses on the individuals of the main analysis. These were conducted as a means of checking of robustness of the results and for effect size modification. (Complementary analysis: 1) We conducted a second set of analyses on a subgroup of individuals born between 1985 and 1999. This time interval allowed us to examine potential confounding in the association between ASD and RA, including, in addition to maternal and paternal age at birth, maternal RA diagnosis, maternal and paternal psychiatric history, and maternal and paternal maximum attained education (to adjust for socioeconomic status at delivery). Missing data on parental education were imputed using the multivariate imputation by chained equations (MICE) method. However, since the missing values are not observed, it is not possible to verify the missing at random (MAR) assumption under MICE. In accordance with the recommendations set forth by the National Research Council, the sensitivity of inferences to departures from the MAR assumption was evaluated through the implementation of a pattern‐mixture model approach under the missing not at random (MNAR) (Molenberghs and Kenward 2007) assumption. We chose the fully conditional specification method (FCS) (National Research Council Panel on Handling Missing Data in Clinical Trials 2010) for an arbitrary missing pattern (number of imputations by SAS default, i.e., 25), which allowed separate conditional distributions for each imputed variable. Missing values for maternal and paternal educational levels were imputed from a discriminant function based on parental income, sex, birth year, parental RA, and parental psychiatric history. (Complementary analysis: 2) We checked in the RA risk varied by offspring sex by examining groups of males and females. We additionally tested for interaction between ASD and sex by adding an interaction term to the adjusted model (model 3).

(Sensitivity analysis: 1) We tested for potential cohort effects by adding an interaction term between birth year and ASD to the model (model 4). (Sensitivity analysis: 2) We implemented a complementary statistical approach that does not make the proportional hazard assumption of the Cox model. Namely, we analyzed the risk by fitting accelerated failure time survival models (AFT) (Wei 1992) with the covariates of birth year and sex. The AFT model analyzes the exposure‐outcome association on the time‐to‐event scale, that is, how much, in relative terms, the time to RA diagnosis is accelerated by ASD diagnosis, but without the potential issues with collapsibility inherited in the Cox regression and thereby allowing the measure of association to be interpreted on a marginal/population level. Three time‐distribution assumptions were assessed by inspecting the Cox–Snell residuals (Cox and Snell 1968): Weibull distribution assumes monotonic hazard and captures early or late failures; log‐normal assumes monotonic or an inverted U‐shaped hazard to convey the initial rise, plateau, and then fall, and predicts around an average time; and log‐logistic assumes decreasing or an inverted bathtub‐shaped hazard and focuses on a gradual rise in failure (Raqab et al. 2018). (Sensitivity analysis: 3) For individuals with ambiguous coding for RA serotype, we restricted the analysis to the most frequent RA diagnosis code over follow‐up. We also performed a fourth analysis where seropositive RA was concluded as soon as any diagnosis of seropositive RA was recorded between the first RA diagnosis and the end of 2018. (Sensitivity analysis: 4) Detailed clinical data on ASD severity or specific features were lacking. Consequently, we examined ASD subtype, non‐Autistic Disorders (AD) ASDs, which represent mild cases respectively, and may partly capture differences.

All analyses were conducted using the SAS software version 9.4 (SAS Institute Inc., Cary, NC, USA). The following statistical procedures were employed: proc. phreg (cox regression), proc. genmod (age‐adjusted rates using Poisson regression), proc. mi (multiple imputation), proc. mianalyze (pooled estimate of multiple imputation), and proc. lifereg (AFT models). All tests of statistical hypotheses were conducted at the two‐sided 5% significance level, corresponding to HRs and AFT estimates lying entirely within the limits of two‐sided 95% CIs.

## 3 Results

The 46,575 individuals diagnosed with ASD were matched to 4,657,500 randomly selected individuals without an ASD diagnosis by sex and age resulting in a cohort of 4,704,075 individuals. Individuals with incomplete maternal or paternal information (23,016, 0.49%) were excluded from the analysis. Thus, the analysis dataset consisted of 4,681,059 individuals (46,164 with ASD and 4,634,895 without ASD), contributing 29,622,644 person‐years of follow‐up for RA (Figure 1). Judged from summary statistics, the individuals with and without ASD had comparable distributions across all matching covariates (Table 1).

**TABLE 1:** Characteristics for individuals with and without an ASD diagnosis.

	Number of individuals diagnosed with ASD (%)	Number of individuals without ASD (%)
Number of individuals (males %)	46,164 (62.66%)	4,634,895 (62.66%)
RA^a^ diagnosis (%, person year)	58 (0.13%, 292,734)	5484 (0.12%, 29,351,910)
Seropositive RA^a^ (%, person year)	25 (0.05%, 292,872)	2517 (0.05%, 29,364,595)
Seronegative RA^a^ (%, person year)	33 (0.07%, 292,840)	2967 (0.06%, 29,363,196)
Birth year		
1952–1961	789 (1.71%)	80,060 (1.73%)
1962–1971	1682 (3.64%)	168,633 (3.64%)
1972–1981	6264 (13.57%)	629,326 (13.58%)
1982–1991	16,305 (35.32%)	1,636,964 (35.32%)
1992–1999	21,124 (45.76%)	2,119,912 (45.73%)
Follow‐up years (median, min–max)	5.62 (0.01–29.71)	5.61 (0.01–30.51)
Follow‐up entry age (median, min–max)	19.24 (18.00–65.68)	19.24 (18.00–65.68)
Follow‐up exit age (median, min–max)	27.63 (18.06–66.96)	27.63 (18.01–66.96)
Maternal age at delivery (years)		
< 20	2122 (4.60%)	149,634 (3.23%)
20–29	26,391 (57.17%)	2,727,098 (58.84%)
30–39	16,450 (35.63%)	1,670,739 (36.05%)
≥ 40	1201 (2.60%)	87,424 (1.90%)
Paternal age at delivery (years)		
< 20	562 (1.22%)	25,658 (0.55%)
20–29	19,624 (42.51%)	2,001,649 (43.19%)
30–39	20,919 (45.31%)	2,186,753 (47.18%)
≥ 40	5059 (10.96%)	410,835 (8.86%)

^a^ RA, specified with ICD‐9 and ICD‐10; Seropositive RA and Seronegative RA, specified with ICD‐10 only; Min, Minimum; Max, Maximum.

During a median follow‐up of 6 years (*Q*1 − Q3 = 3–9), 58 (0.13%) individuals with ASD were diagnosed with RA, of which 25 (0.05%) were diagnosed as seropositive RA and 33 (0.07%) were diagnosed as seronegative RA. In comparison, among individuals without ASD, 5484 (0.12%) were diagnosed with RA of which 2517 (0.05%) were diagnosed as seropositive RA and 2967 (0.06%) as seronegative RA.

### 3.1 ASD and the Risk of RA

The risk of RA among individuals diagnosed with ASD compared to individuals without ASD was estimated at HR = 1.06 (95% CI 0.82–1.37; 19.81 vs. 18.68 RA diagnoses per 100,000 person‐years) for all RA, at HR = 1.00 (95% CI 0.67–1.48; 8.54 vs. 8.57 RA per 100,000 person‐years) for seropositive RA, and at HR = 1.12 (95% CI: 0.79–1.57; 11.27 vs. 10.10 RA per 100,000 person‐years) for seronegative RA. These point estimates were similar after adjustment for maternal and paternal age (Table 2). The age‐specific RA and cumulative incidence of RA are presented in Figure 2 and Figure S2, respectively. There was no evidence of a violation of the proportional hazard assumption (Figure S3).

**TABLE 2:** Hazard ratios for the risk of RA, seronegative RA, and seropositive RA, for individuals diagnosed with and without ASD.

	Number of individuals	RA diagnoses (%)	RA rate per 100,000 person‐years (person years)	Crude model^a^ HR (95% CI)^b^	Adjusted model^c^ HR (95% CI)
RA^b^					
ASD^b^	46,164	58 (0.13%)	19.81 (292,734)	1.06 (0.82–1.37)	1.06 (0.82–1.37)
Without ASD	4,634,895	5484 (0.12%)	18.68 (29,351,910)	Reference	Reference
Seropositive RA^b^					
ASD	46,164	25 (0.05%)	8.54 (292,872)	1.00 (0.67–1.48)	1.00 (0.67–1.48)
Without ASD	4,634,895	2517 (0.05%)	8.57 (29,364,595)	Reference	Reference
Seronegative RA^b^					
ASD	46,164	33 (0.07%)	11.27 (292,840)	1.12 (0.79–1.57)	1.11 (0.79–1.57)
Without ASD	4,634,895	2967 (0.06%)	10.10 (29,363,196)	Reference	Reference

^a^ Crude model: Adjusted for birth year by natural cubic splines and sex.

^b^ Abbreviations: ASD: autism spectrum disorder; HR (95% CI): Hazard ratio with 95% confidence interval; RA: rheumatoid arthritis; Reference: The comparison group.

^c^ Adjusted model: Additionally adjusted for maternal age at delivery (by natural cubic splines and 3 degrees of freedom) and paternal age at delivery (by natural cubic splines and 3 degrees of freedom).

**FIGURE 2:**
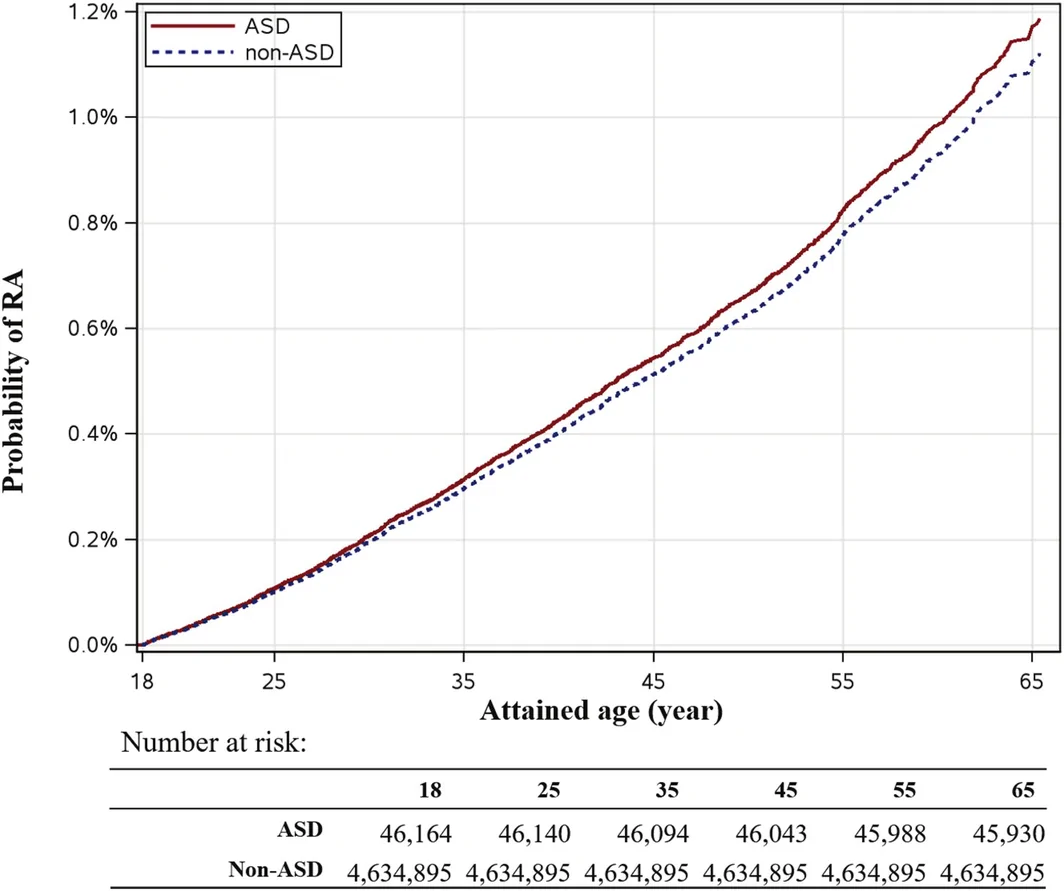
Age specific cumulative probability of rheumatoid arthritis for individuals with and without autism spectrum disorder. Abbreviations: ASD, autism spectrum disorder; RA, rheumatoid arthritis. This figure shows the increased probability of RA along with the age attained for individuals with ASD (solid line) compared to those without ASD (dashed line).

### 3.2 Complementary Analyses

The complementary analysis was conducted on individuals born in 1985 and onwards, and additionally adjusted for maternal RA, maternal and paternal age, psychiatric history, and maximum attained education (to adjust for socioeconomic status), all defined at delivery. The HR was 1.34 (95% CI 0.97–1.84). There was no evidence for confounding by maternal and paternal psychiatric history, maternal and paternal RA, and maternal and paternal education, and there was no support for any differences by RA serostatus. There was no statistically significant fluctuation on the risk estimates alternatively imputed by the pattern‐mixture model approach (Tables S2–S3, Figure S4). There was no evidence to suggest that sex modified the results (Table S4, Figure S5, Supporting Information S1), nor was there any indication of a periodic effect associated with the introduction of ICD‐9 in 1987 (Supporting Information S1). The AFT models further verified the results from the Cox model and estimated the time to RA to occur 6% earlier among individuals with ASD (95% CI: 26% earlier to 21% later) than the expected time to RA among individuals without ASD (Table S5, Figure S6). The results remained null in analyses stratified by alternative definitions of RA subtype (Table S6–S7). The results remained null in analyses stratified by ASD subtypes (Table S8).

## 4 Discussion

Based on a matched national cohort study that included over 4.6 million individuals with up to 30 years of follow‐up, our findings do not indicate an increased risk of developing RA in adulthood among individuals with ASD. The current findings may offer some clinical assurance to the autism community, suggesting that despite well‐established immune dysfunction in ASD, we did not observe a clearly elevated risk for this major autoimmune condition. Nonetheless, these findings should be interpreted with caution, given the limited number of individuals with ASD.

The absence of elevated RA risk in our study contrasts with the established pattern of increased autoimmune comorbidity in ASD, including IBD, psoriasis, and allergic conditions (Zerbo et al. 2015). This differential pattern suggests that immune dysfunction in autism may not uniformly increase risk across all autoimmune diseases. Several biological factors may explain why RA risk is not elevated despite immune dysfunction in ASD. While both conditions share certain genetic variants (particularly HLA‐DRB1*04 alleles) (Johnson et al. 2009), the genetic architecture of each condition is complex and polygenic. Our findings suggest that shared genetic variants alone may be insufficient to confer elevated RA risk in autism populations, but these results should be interpreted with caution given the limited number of exposed cases.

Although the risk of RA was not elevated among individuals with ASD, other aspects of autoimmunity and inflammation may still be relevant to ASD, such as non‐specific inflammatory conditions, subclinical autoimmune activity, or dysregulated cytokine response, which may be relevant to ASD pathophysiology. Additionally, the immune dysregulation patterns in ASD—characterized by elevated pro‐inflammatory cytokines like IL‐1 and TNF—may differ from the specific autoimmune processes that drive RA pathogenesis. In our previous study, we concluded that maternal RA present before delivery, but not after delivery and not for paternal and maternal‐sister RA, is a risk factor for offspring ASD (Yin et al. 2023). These findings suggest alternative pathways for the association between RA and ASD beyond purely genetic association. The maternal‐to‐offspring association likely involves prenatal environmental factors rather than purely genetic transmission, as suggested by Johnson et al.'s findings that maternal HLA‐DRB1*04 alleles increased offspring ASD risk regardless of inheritance (Johnson et al. 2009). Immunogenetic variations in ASD, including differences in HLA alleles and immune regulatory genes (Arenella, Fanelli, et al. 2023; Arenella, Matuleviciute, et al. 2023), may protect against RA while increasing susceptibility of RA through atopic‐like reactions. This heterogeneity in immune mechanisms may partly explain, in our study, the lack of a significant association between ASD and RA risk. In addition, potential mechanisms include maternal cytokine exposure, autoantibodies, medications, or inflammatory processes during critical neurodevelopmental windows. We cannot rule out the possibility that medication, including anti‐inflammatory drugs, plays a role in linking ASD to RA. However, the lack of medication data and the comparably small sample size prevented us from making such assessments. Our null findings for ASD‐to‐RA risk suggest these prenatal environmental factors do not create lasting autoimmune vulnerability in the offspring themselves. This supports autism's clinical heterogeneity and the concept that autism subgroups have distinct medical risk profiles (Masi et al. 2017). Rheumatoid arthritis is an autoimmune disease that we can identify with high reliably in the specialist‐care databases we analyzed. Since the criteria for diagnosing RA are strict and well‐defined, it gives us a clear, “clean” case group to test whether the autoimmune response is different in people with ASD. Finding no increased risk of RA in our ASD cohort doesn't contradict with other studies that report higher rates of immune‐related problems in ASD. Instead, it shows that autism may be linked to other kinds of immune issues such as allergies, gut inflammation, or skin problems that involve an over‐reactive immune system, rather than to the specific self‐directed autoimmunity that characterizes RA.

Our study addresses a critical methodological gap in autism‐autoimmune research. Previous studies examining autoimmune conditions in ASD have been limited by insufficient follow‐up duration to capture adult‐onset diseases like RA, which typically manifests decades after autism diagnosis. Our up to 30‐year follow‐up period, encompassing 29.6 million person‐years of observation, provides unprecedented power to detect associations that shorter studies would miss. Additional strengths include population‐based ascertainment minimizing selection bias, specialist‐confirmed diagnoses enhancing validity, and the ability to examine RA subtypes through Swedish registry ICD coding. The publicly financed healthcare system ensures equal access and comprehensive follow‐up regardless of socioeconomic status.

Several limitations warrant consideration, which also point to important directions for future research. First, unmeasured confounders potentially linked to both ASD and RA risk (Modabbernia et al. 2017), including smoking (Hedström et al. 2018), dietary factors (Johansson et al. 2018), and obesity (Linauskas et al. 2019), were not available in our dataset. Detailed data on ASD severity or clinical features were not available, restricting our ability to assess immune‐related variation across autism phenotypes. Subtype analysis (non‐autistic disorder [non‐AD] ASD) provided preliminary insight but should be interpreted cautiously given the small sample size and wide confidence intervals. Second, the follow‐up period, while unprecedented in autism‐autoimmune research, may still be insufficient to capture the full spectrum of RA risk. Our median age at study end was 28 years, while RA incidence peaks after age of 70. However, our complementary analyses and accelerated failure time models confirmed the robustness of our findings, and the substantial observation period provides strong evidence against major elevation in early‐to‐mid‐adulthood RA risk. Still, extended follow‐up studies examining RA risk in older autism populations would provide valuable insights, particularly as individuals reach peak RA incidence years. Third, healthcare utilization patterns may differ between ASD and non‐ASD populations, potentially affecting diagnosis rates. However, such channeling effects would likely bias toward finding increased RA rates in ASD group, making our null findings more conservative. Fourth, our Swedish population may limit generalizability to other healthcare systems or populations with different genetic backgrounds. International collaborative studies across different healthcare systems and populations would test the generalizability of these findings and support evidence‐based clinical guidelines. Fifth, the limited number of RAs among individuals with ASD (*N* = 58) reduces statistical power and may result in a null association. Similarly, the findings from subgroup analyses were also limited to fewer RA cases in ASDs, and it should be approached with cautious interpretations. Despite a lack of a statistically significant association, the results signal an increased risk. Finally, interpretation of these results should consider the modest number of individuals with ASD and RA, which limits statistical power and precision.

Future studies are warranted to better understand why certain autoimmune conditions show elevated risk in ASD while others do not, which could identify specific immune pathways involved. Examining whether autism severity, intellectual ability, or specific phenotypes modify autoimmune risk patterns would further inform targeted clinical strategies. Although we observed no statistically significant association between ASD and RA, it is important to consider contextual factors that may influence symptom recognition and health‐care‐seeking. Individuals with ASD may experience atypical sensory processing, including altered pain sensitivity, which could affect the perception, interpretation, and reporting of joint pain and thereby delay help‐seeking. This consideration may have implications for equitable access to and responsiveness of clinical services for populations with ASD and warrants further study.

## 5 Conclusion

In this large population‐based study with extended follow‐up, individuals with ASD did not demonstrate increased risk of developing RA compared to matched controls. The findings do not provide evidence for a clinically important association between ASD and subsequent RA. While routine RA screening beyond standard adult care may not be warranted specifically for individuals with ASD, continued research into the specific patterns of medical comorbidity in autism remains essential for optimizing long‐term health outcomes in this population.

## Author Contributions

Sandin had full access to all the data in the study. Sandin and Yip take responsibility for the integrity of the data and the accuracy of the data analysis. Study concept and design: Sandin, Yip, Levine, and Zhu. Acquisition, analysis, or interpretation of data: Sandin, Yip, Levine, and Zhu. Drafting of the manuscript: Levine, Sandin, and Zhu. Critical revision of the manuscript for important intellectual content: Zhu, Levine, Morin, Christoffersen, Askling, Rivera, Reichenberg, Buxbaum, Yin, Yip, and Sandin. Statistical analysis: Zhu. Obtained funding: Sandin, Levine, and Yip. Administrative, technical, or material support: Sandin. Study supervision: Yip, Sandin and Askling.

## Funding

This work was supported by National Institutes of Health (R01AR075812), the Karolinska Institutet's Research Foundation Grants (Dnr 2018‐01499, Dnr 2020‐02156), the European Union's Horizon 2020 award (733280), the Swedish Research Council (Dnr. 2019‐00227), and the Hong Kong Research Grants Council (RGC)—Early Career Scheme (ECS 24108520).

## Ethics Statement

The study was approved by the national Swedish ethics review board, Sweden (2017/1875‐31/2; 2018/1864‐32; 2019‐06314).

## Conflicts of Interest

The authors declare no conflicts of interest.

## Supporting information


**Table S1:** International classification of diseases (ICD) codes used for identification of rheumatoid arthritis (RA), autism spectrum disorder (ASD)
**Table S2:** Individuals characteristics for individuals with ASD and without ASD for individuals born 1985–1999.
**Table S3:** Hazard ratios for the risk of RA, seronegative RA, and seropositive RA, for individuals diagnosed with ASD compared to individuals without ASD in diagnosis for individuals born 1985–1999.
**Table S4:** Hazard ratios for the risk of RA, seronegative RA, and seropositive RA, for males (females) diagnosed with ASD compared to males (females) without ASD diagnosis.
**Table S5:** Acceleration (or deacceleration) of RA, seronegative RA, and seropositive RA for individuals diagnosed with ASD compared to individuals without ASD diagnosis.
**Table S6:** Hazard ratios for the risk of RA, seronegative RA, and seropositive RA, for individuals diagnosed with ASD compared to individuals without ASD diagnosis based on matched cohort, on most frequent RA subtype.
**Table S7:** Hazard ratios for the risk of RA, seronegative RA, and seropositive RA, for individuals diagnosed with ASD compared to individuals without ASD diagnosis based on matched cohort, identified the serostatus by any seropositive RA.
**Table S8:** Hazard ratios for the risk of RA, seronegative RA, and seropositive RA, for individuals diagnosed with and without Non‑AD ASD.
**Figure S1:** Merging Swedish national registers into an analytic dataset for research.
**Figure S2:** Age‐specific RA incidence rate (cases per 100,000 person‐year) among individuals with and without ASD.
**Figure S3:** Weighted Schoenfeld residuals to examine the proportional hazard assumption.
**Figure S4:** Age‐specific cumulative probability of rheumatoid arthritis for individuals with and without ASD, for the subpopulation 1985–1999.
**Figure S5:** Age‐specific cumulative probability of RA for males and females, with and without ASD.
**Figure S6:** The inspection of AFT Cox‐Shell residuals of log‐logistic distribution.
**Supporting Information S1:** Interaction terms: (1) between birth year and ASD; (2) between sex and ASD.

## Data Availability

The data underlying this article cannot be shared publicly due to the ethical requirement for the register data.

## References

[aur70349-bib-0001] Aletaha, D. , and J. S. Smolen . 2018. “Diagnosis and Management of Rheumatoid Arthritis: A Review.” JAMA 320, no. 13: 1360–1372. 10.1001/jama.2018.13103. 30285183

[aur70349-bib-0002] Arenella, M. , G. Fanelli , L. A. Kiemeney , G. McAlonan , D. G. Murphy , and J. Bralten . 2023. “Genetic Relationship Between the Immune System and Autism.” Brain, Behavior, and Immunity—Health 34: 100698. 10.1016/j.bbih.2023.100698. PMC10663755 38020478

[aur70349-bib-0003] Arenella, M. , R. Matuleviciute , R. Tamouza , et al. 2023. “Immunogenetics of Autism Spectrum Disorder: A Systematic Literature Review.” Brain, Behavior, and Immunity 114: 488–499. 10.1016/j.bbi.2023.09.010. 37717669

[aur70349-bib-0004] Atladóttir, H. O. , M. G. Pedersen , P. Thorsen , et al. 2009. “Association of Family History of Autoimmune Diseases and Autism Spectrum Disorders.” Pediatrics 124, no. 2: 687–694. 10.1542/peds.2008-2445. 19581261

[aur70349-bib-0005] Axelsson, O. 2003. “The Swedish Medical Birth Register.” Acta Obstetricia et Gynecologica Scandinavica 82, no. 6: 491–492. 10.1034/j.1600-0412.2003.00172.x 12780418

[aur70349-bib-0006] Breslow, N. E. 1996. “Statistics in Epidemiology: The Case‐Control Study.” Journal of the American Statistical Association 91, no. 433: 14–28. 10.1080/01621459.1996.10476660 12155399

[aur70349-bib-0007] Breslow, N. E. , J. H. Lubin , P. Marek , and B. Langholz . 1983. “Multiplicative Models and Cohort Analysis.” Journal of the American Statistical Association 78, no. 381: 1–12.

[aur70349-bib-0008] Catalá‐López, F. , B. Hutton , M. J. Page , et al. 2022. “Mortality in Persons With Autism Spectrum Disorder or Attention‐Deficit/Hyperactivity Disorder: A Systematic Review and Meta‐Analysis.” JAMA Pediatrics 176, no. 4: e216401. 10.1001/jamapediatrics.2021.6401. PMC8845021 35157020

[aur70349-bib-0009] Chiang, H. L. , C. J. Liu , Y. W. Hu , et al. 2015. “Risk of Cancer in Children, Adolescents, and Young Adults With Autistic Disorder.” Journal of Pediatrics 166, no. 2: 418–423.e411. 10.1016/j.jpeds.2014.10.029. 25453246

[aur70349-bib-0010] Cox, D. R. , and E. J. Snell . 1968. “A General Definition of Residuals.” Journal of the Royal Statistical Society, Series B: Statistical Methodology 30, no. 2: 248–265.

[aur70349-bib-0011] Ekbom, A. 2011. “The Swedish Multi‐Generation Register.” Methods in Biobanking 675: 215–220. 10.1007/978-1-59745-423-0_10 20949391

[aur70349-bib-0012] Ekholm, B. , A. Ekholm , R. Adolfsson , et al. 2005. “Evaluation of Diagnostic Procedures in Swedish Patients With Schizophrenia and Related Psychoses.” Nordic Journal of Psychiatry 59, no. 6: 457–464. 10.1080/08039480500360906 16316898

[aur70349-bib-0013] Eriksson, J. K. , M. Neovius , S. Ernestam , S. Lindblad , J. F. Simard , and J. Askling . 2013. “Incidence of Rheumatoid Arthritis in Sweden: A Nationwide Population‐Based Assessment of Incidence, Its Determinants, and Treatment Penetration.” Arthritis Care and Research (Hoboken) 65, no. 6: 870–878. 10.1002/acr.21900. 23281173

[aur70349-bib-0014] First, M. B. 2013. “Diagnostic and Statistical Manual of Mental Disorders, 5th Edition, and Clinical Utility.” Journal of Nervous and Mental Disease 201, no. 9: 727–729. 10.1097/NMD.0b013e3182a2168a. 23995026

[aur70349-bib-0015] Frome, E. L. , and H. Checkoway . 1985. “Use of Poisson Regression Models in Estimating Incidence Rates and Ratios.” American Journal of Epidemiology 121, no. 2: 309–323. 10.1093/oxfordjournals.aje.a114001 3839345

[aur70349-bib-0016] Hardin, J. W. 2003. “The Sandwich Estimate of Variance.” In Maximum Likelihood Estimation of Misspecified Models: Twenty Years Later, vol. 17, 45–73. Emerald Group Publishing Limited.

[aur70349-bib-0017] Hastie, T. , R. Tibshirani , J. H. Friedman , and J. H. Friedman . 2009. The Elements of Statistical Learning: Data Mining, Inference, and Prediction. Vol. 2. Springer.

[aur70349-bib-0018] He, H. , Y. Yu , Z. Liew , et al. 2022. “Association of Maternal Autoimmune Diseases With Risk of Mental Disorders in Offspring in Denmark.” JAMA Network Open 5, no. 4: e227503‐e227503. 10.1001/jamanetworkopen.2022.7503. PMC9012963 35426923

[aur70349-bib-0019] Hedström, A. K. , L. Stawiarz , L. Klareskog , and L. Alfredsson . 2018. “Smoking and Susceptibility to Rheumatoid Arthritis in a Swedish Population‐Based Case‐Control Study.” European Journal of Epidemiology 33, no. 4: 415–423. 10.1007/s10654-018-0360-5. PMC5945793 29387991

[aur70349-bib-0020] Hughes, H. K. , E. Mills Ko , D. Rose , and P. Ashwood . 2018. “Immune Dysfunction and Autoimmunity as Pathological Mechanisms in Autism Spectrum Disorders.” Frontiers in Cellular Neuroscience 12: 405. 10.3389/fncel.2018.00405. PMC6242891 30483058

[aur70349-bib-0021] Johansson, K. , J. Askling , L. Alfredsson , and D. Di Giuseppe . 2018. “Mediterranean Diet and Risk of Rheumatoid Arthritis: A Population‐Based Case‐Control Study.” Arthritis Research and Therapy 20, no. 1: 175. 10.1186/s13075-018-1680-2. PMC6085628 30092814

[aur70349-bib-0022] Johnson, W. G. , S. Buyske , A. E. Mars , et al. 2009. “HLA‐DR4 as a Risk Allele for Autism Acting in Mothers of Probands Possibly During Pregnancy.” Archives of Pediatrics and Adolescent Medicine 163, no. 6: 542–546. 10.1001/archpediatrics.2009.74. 19487610

[aur70349-bib-0023] Kahathuduwa, C. N. , B. D. West , J. Blume , N. Dharavath , N. Moustaid‐Moussa , and A. Mastergeorge . 2019. “The Risk of Overweight and Obesity in Children With Autism Spectrum Disorders: A Systematic Review and Meta‐Analysis.” Obesity Reviews 20, no. 12: 1667–1679. 10.1111/obr.12933. 31595678

[aur70349-bib-0024] Kurkó, J. , T. Besenyei , J. Laki , T. T. Glant , K. Mikecz , and Z. Szekanecz . 2013. “Genetics of Rheumatoid Arthritis ‐ a Comprehensive Review.” Clinical Reviews in Allergy and Immunology 45, no. 2: 170–179. 10.1007/s12016-012-8346-7. PMC3655138 23288628

[aur70349-bib-0025] Lee, M. , J. Krishnamurthy , A. Susi , et al. 2018. “Association of Autism Spectrum Disorders and Inflammatory Bowel Disease.” 48: 1523–1529. 10.1007/s10803-017-3409-5 29170940

[aur70349-bib-0026] Linauskas, A. , K. Overvad , D. Symmons , M. B. Johansen , K. Stengaard‐Pedersen , and A. de Thurah . 2019. “Body Fat Percentage, Waist Circumference, and Obesity as Risk Factors for Rheumatoid Arthritis: A Danish Cohort Study.” Arthritis Care and Research (Hoboken) 71, no. 6: 777–786. 10.1002/acr.23694. 29975015

[aur70349-bib-0027] Ludvigsson, J. F. , C. Almqvist , A.‐K. E. Bonamy , et al. 2016. “Registers of the Swedish Total Population and Their Use in Medical Research.” European Journal of Epidemiology 31: 125–136. 10.1007/s10654-016-0117-y 26769609

[aur70349-bib-0028] Ludvigsson, J. F. , P. Svedberg , O. Olén , G. Bruze , and M. Neovius . 2019. “The Longitudinal Integrated Database for Health Insurance and Labour Market Studies (LISA) and Its Use in Medical Research.” European Journal of Epidemiology 34, no. 4: 423–437. 10.1007/s10654-019-00511-8. PMC6451717 30929112

[aur70349-bib-0029] Ly, A. , B. Leppert , D. Rai , H. Jones , C. Dardani , and E. Stergiakouli . 2022. “Genetic Liability to Rheumatoid Arthritis on Autism and Autistic Traits: Polygenic Risk Score and Mendelian Randomization Analyses.” Translational Psychiatry 12, no. 1: 18. 10.1038/s41398-021-01772-2. PMC8755835 35022383

[aur70349-bib-0030] Masi, A. , M. M. DeMayo , N. Glozier , and A. J. Guastella . 2017. “An Overview of Autism Spectrum Disorder, Heterogeneity and Treatment Options.” Neuroscience Bulletin 33, no. 2: 183–193. 10.1007/s12264-017-0100-y. PMC5360849 28213805

[aur70349-bib-0031] Meltzer, A. , and J. Van de Water . 2017. “The Role of the Immune System in Autism Spectrum Disorder.” Neuropsychopharmacology 42, no. 1: 284–298. 10.1038/npp.2016.158. PMC5143489 27534269

[aur70349-bib-0032] Modabbernia, A. , E. Velthorst , and A. Reichenberg . 2017. “Environmental Risk Factors for Autism: An Evidence‐Based Review of Systematic Reviews and Meta‐Analyses.” Molecular Autism 8: 13. 10.1186/s13229-017-0121-4. PMC5356236 28331572

[aur70349-bib-0033] Molenberghs, G. , and M. Kenward . 2007. Missing Data in Clinical Studies. John Wiley & Sons.

[aur70349-bib-0034] National Research Council Panel on Handling Missing Data in Clinical Trials . 2010. The Prevention and Treatment of Missing Data in Clinical Trials. National Academies Press. Copyright 2010 by the National Academy of Sciences. All rights reserved. 10.17226/12955.

[aur70349-bib-0035] Neovius, M. , J. F. Simard , J. Askling , and ARTIS Study Group . 2011. “Nationwide Prevalence of Rheumatoid Arthritis and Penetration of Disease‐Modifying Drugs in Sweden.” Annals of the Rheumatic Diseases 70, no. 4: 624–629. 10.1136/ard.2010.133371 21149495

[aur70349-bib-0036] Nieto, F. J. , and J. Coresh . 1996. “Adjusting Survival Curves for Confounders: A Review and a New Method.” American Journal of Epidemiology 143, no. 10: 1059–1068. 10.1093/oxfordjournals.aje.a008670 8629613

[aur70349-bib-0037] Nørgaard, M. , H. Larsson , L. Pedersen , et al. 2010. “Rheumatoid Arthritis and Birth Outcomes: A Danish and Swedish Nationwide Prevalence Study.” Journal of Internal Medicine 268, no. 4: 329–337. 10.1111/j.1365-2796.2010.02239.x 20456595

[aur70349-bib-0038] Quan, H. , B. Li , L. Duncan Saunders , et al. 2008. “Assessing Validity of ICD‐9‐CM and ICD‐10 Administrative Data in Recording Clinical Conditions in a Unique Dually Coded Database.” Health Services Research 43, no. 4: 1424–1441. 10.1111/j.1475-6773.2007.00822.x PMC2517283 18756617

[aur70349-bib-0039] Raqab, M. Z. , S. A. Al‐Awadhi , D. J. C. i. S.‐S. Kundu , and Computation . 2018. “Discriminating Among Weibull, Log‐Normal, and Log‐Logistic Distributions.” 47, no. 5: 1397–1419.

[aur70349-bib-0040] Saad, K. , A. M. Abdallah , A. A. Abdel‐Rahman , et al. 2020. “Polymorphism of Interleukin‐1β and Interleukin‐1 Receptor Antagonist Genes in Children With Autism Spectrum Disorders.” Progress in Neuro‐Psychopharmacology and Biological Psychiatry 103: 109999. 10.1016/j.pnpbp.2020.109999. 32526258

[aur70349-bib-0041] Sandin, S. , D. Schendel , P. Magnusson , et al. 2016. “Autism Risk Associated With Parental Age and With Increasing Difference in Age Between the Parents.” Molecular Psychiatry 21, no. 5: 693–700. 10.1038/mp.2015.70. PMC5414073 26055426

[aur70349-bib-0042] Symmons, D. , G. Turner , R. Webb , et al. 2002. “The Prevalence of Rheumatoid Arthritis in the United Kingdom: New Estimates for a New Century.” Rheumatology (Oxford, England) 41, no. 7: 793–800. 10.1093/rheumatology/41.7.793. 12096230

[aur70349-bib-0043] Torres, A. R. , J. B. Westover , and A. J. Rosenspire . 2012. “HLA Immune Function Genes in Autism.” Autism Research and Treatment 2012: 959073. 10.1155/2012/959073. PMC3420779 22928105

[aur70349-bib-0044] Uthman, O. A. 2016. “Global, Regional, National Incidence, Prevalence and Years Lived With Disability for 310 Acute and Chronic Diseases and Injuries 1990–2015: A Systematic Analysis for the Global Burden of Disease Study 2015.” Lancet 388, no. 10053: 1545–1602. 10.1016/S0140-6736(16)31678-6 PMC5055577 27733282

[aur70349-bib-0045] Vivanti, G. , S. Tao , K. Lyall , D. L. Robins , and L. L. Shea . 2021. “The Prevalence and Incidence of Early‐Onset Dementia Among Adults With Autism Spectrum Disorder.” Autism Research 14, no. 10: 2189–2199. 10.1002/aur.2590. PMC8487995 34378867

[aur70349-bib-0046] Waldenlind, K. , J. K. Eriksson , B. Grewin , and J. Askling . 2014. “Validation of the Rheumatoid Arthritis Diagnosis in the Swedish National Patient Register: A Cohort Study From Stockholm County.” BMC Musculoskeletal Disorders 15: 432. 10.1186/1471-2474-15-432. PMC4302140 25510838

[aur70349-bib-0047] Watson, D. J. , T. Rhodes , and H. A. Guess . 2003. “All‐Cause Mortality and Vascular Events Among Patients With Rheumatoid Arthritis, Osteoarthritis, or No Arthritis in the UK General Practice Research Database.” Journal of Rheumatology 30, no. 6: 1196–1202. 12784389

[aur70349-bib-0048] Wei, L.‐J. 1992. “The Accelerated Failure Time Model: A Useful Alternative to the Cox Regression Model in Survival Analysis.” Statistics in Medicine 11, no. 14–15: 1871–1879. 10.1002/sim.4780111409 1480879

[aur70349-bib-0049] Xie, J. , L. Huang , X. Li , et al. 2017. “Immunological Cytokine Profiling Identifies TNF‐α as a Key Molecule Dysregulated in Autistic Children.” Oncotarget 8, no. 47: 82390–82398. 10.18632/oncotarget.19326 PMC5669898 29137272

[aur70349-bib-0050] Yin, W. , M. Norrbäck , S. Z. Levine , et al. 2023. “Maternal Rheumatoid Arthritis and Risk of Autism in the Offspring.” Psychological Medicine 53, no. 15: 7300–7308. 10.1017/S0033291723000855. PMC10593909 37092864

[aur70349-bib-0051] Zerbo, O. , A. Leong , L. Barcellos , P. Bernal , B. Fireman , and L. A. Croen . 2015. “Immune Mediated Conditions in Autism Spectrum Disorders.” Brain, Behavior, and Immunity 46: 232–236. 10.1016/j.bbi.2015.02.001. PMC4414798 25681541

[aur70349-bib-0052] Zhu, E. H. , B. H. Yip , C. Fyfe , et al. 2025. “Maternal Rheumatoid Arthritis and the Risk of Offspring Autism Spectrum Disorder: Two National Birth Cohorts and a Meta‐Analysis.” Molecular Autism 16, no. 1: 61. 10.1186/s13229-025-00694-w. PMC12679756 41345879

[aur70349-bib-0053] Zhu, Z. , S. Tang , X. Deng , and Y. Wang . 2020. “Maternal Systemic Lupus Erythematosus, Rheumatoid Arthritis, and Risk for Autism Spectrum Disorders in Offspring: A Meta‐Analysis.” Journal of Autism and Developmental Disorders 50, no. 8: 2852–2859. 10.1007/s10803-020-04400-y. 32034648

