# Supplementary material for: Long‐Term Risk of Rheumatoid Arthritis in Individuals With Autism Spectrum Disorder: A Population‐Based Matched Cohort Study
Source: Autism Res. 2026 Aug 28;19(10):e70349. doi: 10.1002/aur.70349 (PMC13674361; doi:10.1002/aur.70349)
Supplement: Supplementary file 1 [file AUR-19-0-s001.pdf]

List of contents Page

Appendix:

Supplementary Table S1: International Classification of Diseases (ICD) codes used for identification of Rheumatoid Arthritis (RA), Autism Spectrum Disorder (ASD)....2

Supplementary Table S2 Individuals characteristics for individuals with ASD and without ASD for individuals born 1985 – 1999....3

Supplementary Table S3: Hazard Ratios for the risk of RA, Seronegative RA, and Seropositive RA, for individuals diagnosed with ASD compared to individuals without ASD in diagnosis for individuals born 1985 – 1999....4

Supplementary Table S4: Hazard Ratios for the risk of RA, Seronegative RA and Seropositive RA, for males (females) diagnosed with ASD compared to males (females) without ASD diagnosis....5

Supplementary Table S5: Acceleration (or deacceleration) of RA, Seronegative RA and Seropositive RA for individuals diagnosed with ASD compared to individuals without ASD diagnosis....6

Supplementary Table S6: Hazard Ratios for the risk of RA, Seronegative RA and Seropositive RA, for individuals diagnosed with ASD compared to individuals without ASD diagnosis based on matched cohort, on most frequent RA subtype....7

Supplementary Table S7: Hazard Ratios for the risk of RA, Seronegative RA and Seropositive RA, for individuals diagnosed with ASD compared to individuals without ASD diagnosis based on matched cohort, identified the serostatus by any seropositive RA....8

Supplementary Table S8: Hazard Ratios for the risk of RA, Seronegative RA and Seropositive RA, for individuals diagnosed with and without Non-AD ASD....9

Supplementary Figure S1: Merging Swedish national registers into an analytic dataset for researrch 10

Supplementary Figure S2: Age-specific RA incidence rate (cases per 100,000 person-year) among individuals with and without ASD....11

Supplementary Figure S3: Weighted Schoenfeld residuals to examine the proportional hazard assumption....12

Supplementary Figure S4: Age-specific cumulative probability of Rheumatoid Arthritis for individuals with and without ASD, for the subpopulation 1985 – 1999....13

Supplementary Figure S5: Age-specific cumulative probability of RA for males and females, with and without ASD....14

Supplementary Figure S6: The inspection of AFT Cox-Shell residuals of log-logistic distribution. 15

Supplementary data S1: Interaction terms: (1) between birth year and ASD; (2) between sex and ASD....16

eReference....17

.

Supplementary Table S1: International Classification of Diseases (ICD) codes used for identification of Rheumatoid Arthritis (RA), Autism Spectrum Disorder (ASD)

Diagnosis	ICD 10*	ICD 9 (Swedish version)
**Rheumatoid Arthritis**	M05, M06	714A, 714B, 714C, 714W
Seropositive RA	M05	714A, 714B, 714C
Seronegative RA	M06	714W
**Autism Spectrum Disorder**	F84.0, F84.1, F84.3, F84.5, F84.8, F84.9	299A

*ICD 9/10: International Classification of Diseases, version9/version 10[1,2]

Supplementary Table S2 Individuals characteristics for individuals with ASD and without ASD for individuals born 1985 – 1999

	Individuals diagnosed with ASD N (%)	Individuals not diagnosed with ASD N (%)
Number of individuals (males %)	31,831 (64.16%)	3,198,291 (64.23%)
RA cases (%, person-year)	37 (0.12%, 177,433)	2,575 (0.08%, 17,819,046)
Seropositive RA (%, person-year)	15 (0.05%, 177,507)	1,111 (0.10%, 17,823,157)
Seronegative RA (%, person-year)	22 (0.07%, 177,511)	1,464 (0.05%, 17,824,469)
Birth year		
1985 - 1989	6,948 (21.83%)	708,908 (22.17%)
1990 – 1994	12,107 (38.04%)	1,209,041 (37.80%)
1995 – 1999	12,776 (40.14%)	1,280,342 (40.03%)
Follow-up years (median, min - max)	4.98 (0.03 - 15.96)	4.96 (0.00- 16.00)
Maternal age at delivery (years)		
<20	1,080 (3.39%)	63,115 (1.97%)
20-29	17,741 (55.73%)	1,810,360 (56.60%)
30-39	12,121 (38.08%)	1,258,741 (39.36%)
>=40	889 (2.79%)	66,075 (2.07%)
Paternal age at delivery (years)		
<20	327 (1.03%)	16,356 (0.51%)
20-29	12,833 (40.32%)	1,287,939 (40.27%)
30-39	14,991 (47.10%)	1,587,776 (49.64%)
>=40	3,680 (11.56%)	306,220 (9.57%)
Maternal psychiatric history	9,071 (28.50%)	448,700 (14.03%)
Paternal psychiatric history	6,949 (21.83%)	394,393 (12.33%)
Maternal RA	494 (1.55%)	40,780 (1.28%)
Paternal RA	179 (0.56%)	18,968 (0.59%)
Maternal educational > 9 years	31,311 (98.37%)	3,140,543 (98.19%)
Paternal educational > 9 years	30,785 (96.71%)	3,102,150 (96.99%)

1. RA: specified with ICD-9 & ICD-10; Seropositive RA & Seronegative RA: specified with ICD-10 only; ASD: Autism Spectrum Disorder; Min: Minimum; Max: Maximum;

Supplementary Table S3: Hazard Ratios for the risk of RA, Seronegative RA, and Seropositive RA, for individuals diagnosed with ASD compared to individuals without ASD in diagnosis for individuals born 1985 – 1999

	Number of subjects	RA cases (%)	RA rate/100,000 person-years (person years)	Crude model[2] HR (95% CI)	Adjusted model 1[3] HR (95% CI)	Adjusted model 2[4] HR (95% CI)	Adjusted model 3[5] HR (95% CI)
**RA[1]:**							
ASD[1]	34,215	38 (0.11%)	19.62 (193,716)	1.36 (0.99 - 1.88)	1.37 (0.99 – 1.88)	1.34 (0.97 – 1.84)	1.34 (0.97 – 1.84)
non-ASD	3,434,104	2,785 (0.08%)	14.37 (19,382,699)	Reference	Reference	Reference	Reference
**Seropositive RA[1]:**							
ASD	34,215	15 (0.04%)	7.74 (193,801)	1.25 (0.75 – 2.08)	1.25 (0.75 – 2.09)	1.19 (0.72 – 1.99)	1.19 (0.71 – 1.98)
non-ASD	3,434,104	1,197 (0.03%)	6.17 (19,388,721)	reference	Reference	Reference	Reference
**Seronegative RA[1]:**							
ASD	34,215	23 (0.07%)	11.87 (193,789)	1.45 (0.96 – 2.18)	1.45 (0.96 – 2.19)	1.45 (0.96 – 2.20)	1.45 (0.96 – 2.20)
non-ASD	3,434,104	1,588 (0.05%)	8.19 (19,387,148)	Reference		Reference	Reference

1. Abbreviations, RA: Rheumatoid arthritis; ASD: Autism Spectrum Disorder; reference: The comparison group; HR(95% CI): Hazard Ratio with 95% Confidence interval;

2. Crude model: Adjusted for birth year by natural cubic splines with 3 degrees of freedom and sex

3. Adjusted model 1: Additionally adjusted for maternal age at delivery (by natural cubic splines with 3 degrees of freedom) and paternal age at delivery (by natural cubic splines with 3 degrees of freedom)

4. Adjusted model 2: based on adjusted model 1, additionally adjusted for maternal psychiatric history, paternal psychiatric history, maternal RA, and paternal RA

5. Adjusted model 3: based on adjusted model 2, additionally adjusted for maternal educational levels above 9 years (yes/no) and paternal educational levels above 9 years (yes/no)

6. There was no statistically significant fluctuation of HR in adjusted model 3, alternatively imputed by pattern-mixture model approach for all-cause RA (HR=1.34, 95% CI 0.97 - 1.85), seropositive RA (HR=1.19, 95% CI 0.71 – 1.98), or seronegative RA (HR=1.45, 95% CI: 0.96-2.20)

Supplementary Table S4: Hazard Ratios for the risk of RA, Seronegative RA and Seropositive RA, for males (females) diagnosed with ASD compared to males (females) without ASD diagnosis

RA overall and by serostatus	***Sex***	***ASD or non-ASD***	Number of subjects	RA cases (%)	RA rate per 100,000 person-years (person years)	Crude model[3] HR (95% CI)[1]	p-value	Adjusted model[4] HR (95% CI)	p-value
RA[1]:	Male	ASD[1]	28,925	18 (0.06%)	9.66 (180,378)	1.08 (0.68 - 1.71)	0.76	1.07 (0.67 - 1.70)	0.79
		non-ASD	2,904,156	1,693 (0.06%)	9.04 (18,722,439)	Reference		Reference	
	Female	ASD	17,239	40 (0.23%)	37.61 (106,356)	1.06 (0.77 - 1.44)	0.74	1.06 (0.78 - 1.45)	0.72
		non-ASD	1,730,739	3,791 (0.20 %)	35.67 (10,629,471)	Reference		Reference	
**Seropositive RA[1]:**	Male	ASD	28,925	5 (0.02%)	2.68 (186,429)	0.69 (0.29 - 1.66)	0.40	0.68 (0.28 - 1.65)	0.40
		non-ASD	2,904,156	736 (0.03%)	3.93 (18,726,635)	Reference		Reference	
	Female	ASD	17,239	20 (0.12%)	18.79 (106,442)	1.12 (0.72 - 1.75)	0.60	1.13 (0.73 - 1.75)	0.59
		non-ASD	1,730,739	1,781 (0.10%)	16.74 (10,637,960)	Reference		Reference	
**Seronegative RA[1]:**	Male	ASD	28,925	13 (0.04%)	6.97 (186,398)	1.38 (0.80 - 2.38)	0.25	1.36 (0.79 -2.35)	0.27
		non-ASD	2,904,156	957 (0.03%)	5.11 (18,725,842)	Reference		Reference	
	Female	ASD	17,239	20 (0.12%)	18.79 (106,442)	1.00 (0.64 - 1.55)	0.98	1.00 (0.64 - 1.55)	0.99
		non-ASD	1,730,739	2,010 (0.12%)	18.90 (10,637,354)	Reference		Reference	

1. Abbreviations RA: Rheumatoid arthritis; ASD: Autism Spectrum Disorder; reference: The comparison group; HR (95% CI): Hazard Ratio with 95% Confidence interval; P-value: P-value for the test of null hypothesis that HR = 1, which indicates RA is not significantly (P>.05) associated with ASD.

2. Crude model: Adjusted for birth year by natural cubic splines with 3 degrees of freedom and sex

3. Adjusted model 1: Additionally adjusted for maternal age at delivery (by natural cubic splines with 3 degrees of freedom) and paternal age at delivery (by natural cubic splines with 3 degrees of freedom)

Supplementary Table S5: Acceleration (or deacceleration) of RA, Seronegative RA and Seropositive RA for individuals diagnosed with ASD compared to individuals without ASD diagnosis

	Number of subjects	RA cases (%)	RA rate per 100,000 person-years (person years)	Time to RA[3] Median (95%CI)	Weibull[4] Acceleration[5] (95% CI)	AIC[6]	Lognormal Acceleration (95% CI)	AIC	Log-logistic Acceleration (95% CI)	AIC
Outcome: RA[1]										
ASD[1]	46,164	58 (0.13%)	19.81 (292,734)	3.7 (2.9 - 4.8)	0.95 (0.74- 1.21)	90,166	0.93 (0.71- 1.21)	90,228	0.94 (0.74- 1.21)	90,165
non-ASD	4,634,895	5,484 (0.12%)	18.68 (29,351,910)	4 (3.4 - 4.7)	Reference		Reference		Reference	
Seropositive RA[1]										
ASD	46,164	25 (0.05%)	8.54 (292,872)	4 (2.7 - 5.8)	1.00 (0.79 - 1.47)	44,988	1.00 (0.67 - 1.49)	45,008	1.00 (0.69 - 1.46)	44,988
non-ASD	4,634,895	2,517 (0.05%)	8.57 (29,364,595)	4 (3.1 - 5.2)	Reference				Reference	
Seronegative RA[1]										
ASD	46,164	33 (0.07%)	11.27 (292,840)	3.5 (2.5 – 5.0)	0.90 (0.64 - 1.26)	52,822	0.88 (0.61 - 1.25)	52,849	0.90 (0.64 - 1.26)	52,822
non-ASD	4,634,895	2,967 (0.06%)	10.10 (29,363,196)	3.9 (3.1 – 5.0)	Reference		Reference		Reference	

1. Abbreviations. RA: Rheumatoid arthritis; ASD: Autism Spectrum Disorder; reference: The comparison group; HR(95% CI): Hazard Ratio with 95% Confidence interval.
2. Time to RA: Time to RA after ASD onset. For non-ASD group was the summarized statistics with the 95% confidence interval under normal distribution assumption; for the ASD group was the estimation based on the acceleration factor.

3. AFT models are fitted for each RA outcome above (seronegative and seropositive and combined). We compared Weibull, log-normal and log-logistic distributions and selected the most parsimonious model based on the smallest AIC value. Covariates were birth year and the acceleration (or deacceleration) is estimated as the exponential coefficient of ASD status. Weibull distribution assumes monotonic hazard and captures early or late failures; log-normal assumes monotonic or an inverted bathtub shaped hazard and predicts around an average time; and log-logistic assumes decreasing or an inverted bathtub shaped hazard and focuses on a gradual rise in failure[3]

4. Acceleration: An acceleration of less than one corresponds to accelerated RA development for individuals diagnosed with ASD compared to ASD-free individuals. Interpretation illustration: For individuals with ASD, if the acceleration is estimated at 0.94 (0.74- 1.21), taking 1.00 (100%) as the reference level. The time of ASDs to develop RA was accelerated at 94% (74% - 121%) of the time of non-ASDs. In other words, RA is expected to occur 6% (95% CI: 26% earlier to 21% delayed) earlier compared to individuals without ASD.

5. AIC: Akaike information criterion used to select the most appropriate distribution. Here we fit the AIC statistics of the logged response of survival time.

Supplementary Table S6: Hazard Ratios for the risk of RA, Seronegative RA and Seropositive RA, for individuals diagnosed with ASD compared to individuals without ASD diagnosis based on matched cohort, on most frequent RA subtype

	Number of subjects	RA cases (%)	RA rate/100,000 person-years (person years)	Crude model[3] HR (95% CI)[1]	Adjusted model[4] HR (95% CI)
RA[1]:					
ASD[1]	46,164	58 (0.13%)	19.81 (292,734)	1.06 (0.82 – 1.37)	1.06 (0.82 – 1.37)
non-ASD	4,634,895	5,484 (0.12%)	18.68 (29,352,118)	Reference	Reference
Seropositive RA[1]:					
ASD	46,164	25 (0.05%)	8.54 (292,872)	1.01 (0.68 – 1.50)	1.01 (0.68 - 1.50)
non-ASD	4,634,895	2,475 (0.05%)	8.43 (29,365,024)	Reference	Reference
Seronegative RA[1]:					
ASD	46,164	33 (0.07%)	11.27 (292,840)	1.10 (0.78 - 1.55)	1.10 (0.78 - 1.55)
non-ASD	4,634,895	3,009 (0.06%)	10.25 (29,362,984)	Reference	Reference

1. Abbreviations RA: Rheumatoid arthritis; ASD: Autism Spectrum Disorder; Reference: The comparison group; HR (95% CI): Hazard Ratio with 95% Confidence interval

2. Crude model: Adjusted for birth year by natural cubic splines with 3 degrees of freedom and sex

3. Adjusted model 1: Additionally adjusted for maternal age at delivery (by natural cubic splines with 3 degrees of freedom) and paternal age at delivery (by natural cubic splines with 3 degrees of freedom)

Supplementary Table S7: Hazard Ratios for the risk of RA, Seronegative RA and Seropositive RA, for individuals diagnosed with ASD compared to individuals without ASD diagnosis based on matched cohort, identified the serostatus by any seropositive RA

	Number of subjects	RA cases (%)	RA rate/100,000 person-years (person years)	Crude model[3] HR (95% CI)[1]	Adjusted model[4] HR (95% CI)
RA[1]:					
ASD[1]	46,164	58 (0.13%)	19.81 (292,741)	1.06 (0.82 – 1.37)	1.06 (0.82 – 1.37)
non-ASD	4,634,895	5,484 (0.12%)	18.68 (29,352,668)	Reference	Reference
Seropositive RA[1]:					
ASD	46,164	31 (0.07%)	10.59 (292,862)	1.05 (0.74 – 1.50)	1.05 (0.74 - 1.50)
non-ASD	4,634,895	2,955 (0.06%)	10.06 (29,362,691)	Reference	Reference
Seronegative RA[1]:					
ASD	46,164	27 (0.06%)	9.22 (292,857)	1.07 (0.73 - 1.57)	1.07 (0.73 - 1.56)
non-ASD	4,634,895	2,529 (0.05%)	8.61 (29,365,868)	Reference	Reference

1. RA: Rheumatoid arthritis; ASD: Autism Spectrum Disorder; reference: The comparison group; HR(95% CI): Hazard Ratio with 95% Confidence interval; any seropositive RA: this patient was diagnosed with seropositive RA once he/she got the positive results in the blood test regardless of the previous or following negative results

2. Crude model: Adjusted for birth year by natural cubic splines with 3 degrees of freedom and sex

3. Adjusted model 1: Additionally adjusted for maternal age at delivery (by natural cubic splines with 3 degrees of freedom) and paternal age at delivery (by natural cubic splines with 3 degrees of freedom)

Supplementary Table S8: Hazard Ratios for the risk of RA, Seronegative RA and Seropositive RA, for individuals diagnosed with and without Non-AD ASD

	Number of subjects	RA cases (%)	RA rate/100,000 person-years (person years)	Crude model[3] HR (95% CI)[1]	Adjusted model[4] HR (95% CI)
RA[1]:					
Non-AD ASD[1]	36,402	53 (0.15%)	23.22 (228,258)	1.19 (0.91 – 1.56)	1.18 (0.90 – 1.55)
Matched controls	3,656,275	4,493 (0.12%)	19.59 (22,930,187)	Reference	Reference
Seropositive RA[1]:					
Non-AD ASD[1]	36,402	30 (0.08%)	13.14 (228,359)	1.16 (0.78 – 1.74)	1.16 (0.78 – 1.74)
Matched controls	3,656,275	2,074 (0.07%)	10.96 (22,937,593)	Reference	Reference
Seronegative RA[1]:					
Non-AD ASD[1]	36,402	23 (0.06%)	10.07 (228,369)	1.21 (0.84 – 1.74)	1.20 (0.83 – 1.74)
Matched controls	3,656,275	2,416 (0.05%)	8.63 (22,941,053)	Reference	Reference

1. Abbreviations. RA: Rheumatoid arthritis; Non-AD ASD: the rest spectrum disorders except Autistic Disorder in ASD, generally the mild subtypes; reference: The comparison group; HR(95% CI): Hazard Ratio with 95% Confidence interval

2. Crude model: Adjusted for birth year by natural cubic splines and sex

3. Adjusted model: Additionally adjusted for maternal age at delivery (by natural cubic splines and 3 degrees of freedom) and paternal age at delivery (by natural cubic splines and 3 degrees of freedom)

Supplementary Figure S1: Merging Swedish national registers into an analytic dataset for researrch

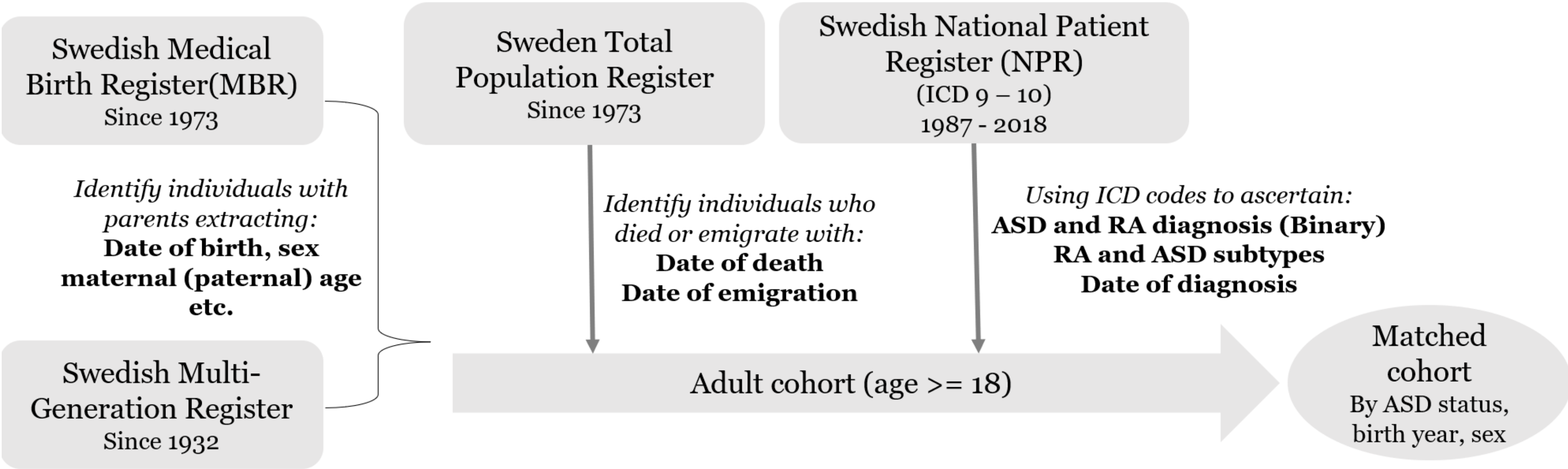

Footnote. The source population was identified in the Swedish Medical Birth Register (MBR) which covers 99% of all birth information nationwide since 1973. Information to link families was identified from the Swedish Multi-Generation Register that is up to date since 1932. After combining these two registers, we constructed the adult cohort born before 1999(age >=18, follow-up RA for at least one year) together with their parental birth information. We then linked death and emigrating information from Sweden Total Population Register, both available from 1973, with the censoring information of date of death and date of emigration. Next, we linked the diagnostic information on ICD-9 and ICD-10 since 1987 from the National Patient Register (NPR). With the organized birth cohort (i.e. the merged registries above), we implemented random matching by ASD status, birth year, and sex. Finally, we constructed the analytical matched cohort.

Supplementary Figure S2: Age-specific RA incidence rate (cases per 100,000 person-year) among individuals with and without ASD

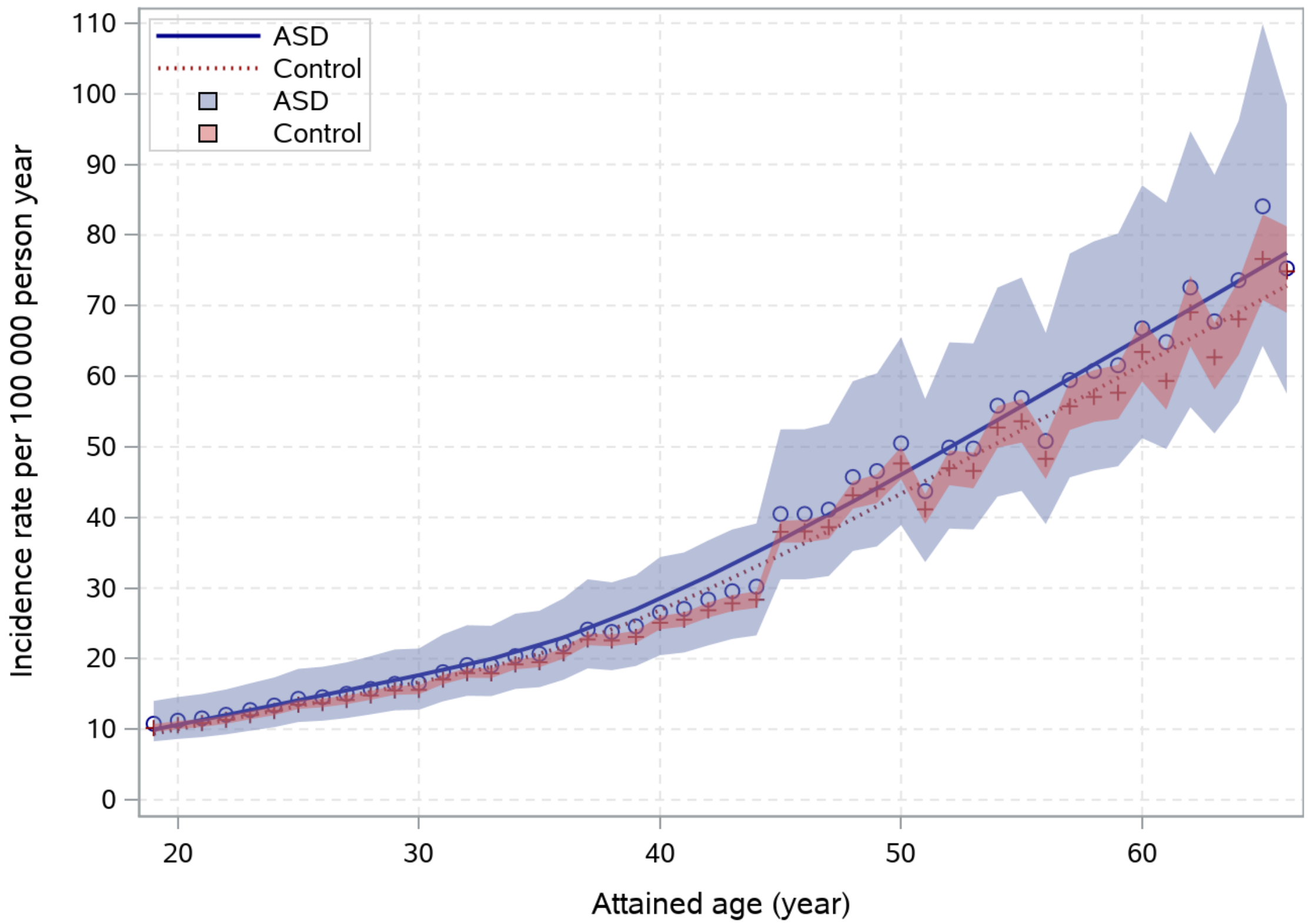

Footnote. Two fitted curves of adjusted incidence rate with 95% confidence intervals (95%CI): one for individuals with ASD (blue solid line with the corresponding band of 95% CI) and one for those without ASD (Control, red solid line with the corresponding band of 95% CI). The circles represent the mean predicted incidence rates of ASDs (blue) and non-ASDs (red). The plot is based on Poisson regression adjusted for age, sex (male/female, female as the reference level) and with an offset parameter for the person-years at risk in each age-sex-cell. The x-axis represents the age, which is calculated as the age attained at the end of follow-up. The y-axis represents the age-sex-adjusted incidence rate.

Supplementary Figure S3: Weighted Schoenfeld residuals to examine the proportional hazard assumption

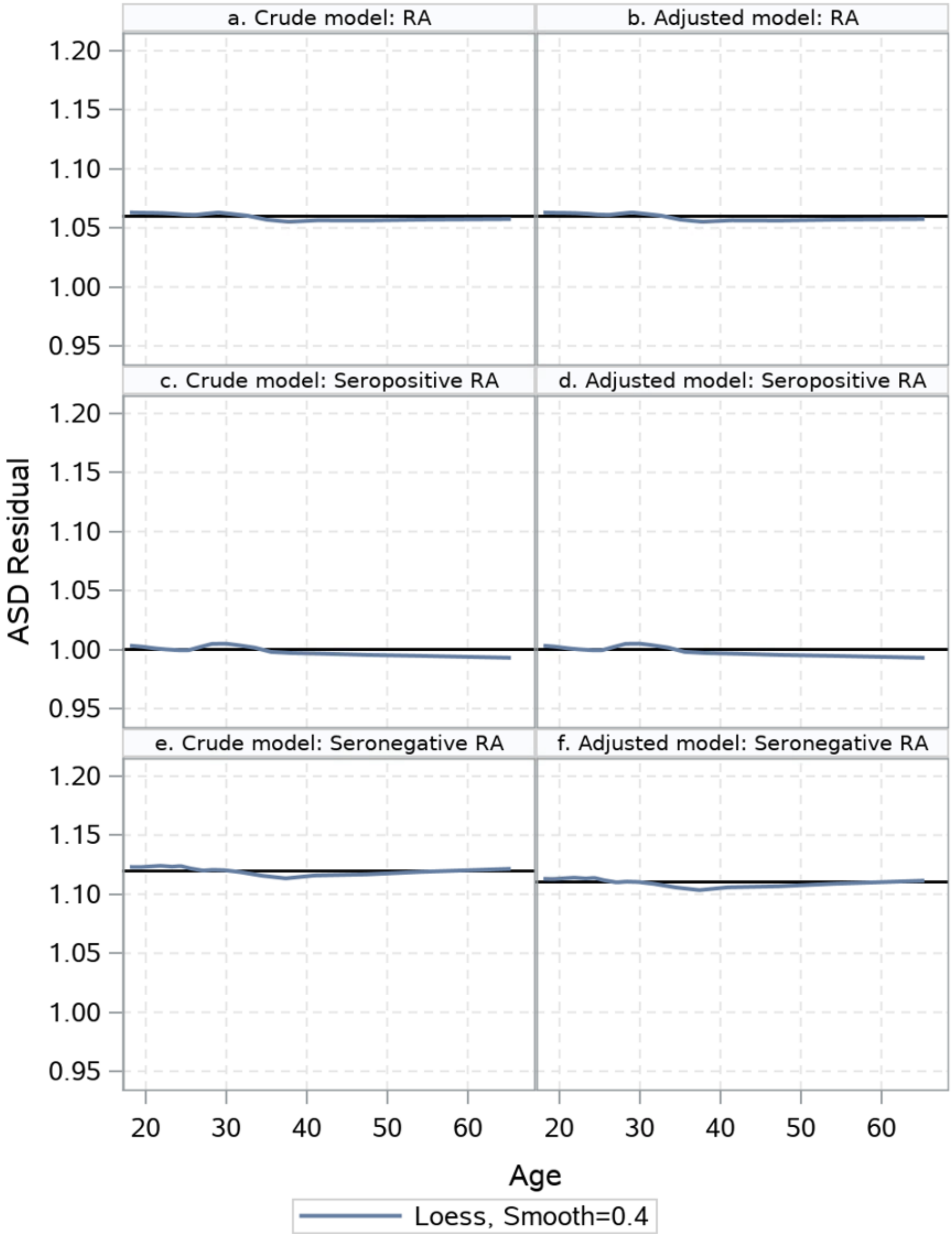

Footnote. Examine the assumption of proportional hazard of the cox models in **Table2** of the manuscript. The cox models calculated the relative risk (hazard ratio) of RA and subtypes (seropositive RA and seronegative RA) in ASD individuals. The crude model adjusted for the birth year by natural cubic splines and sex. The adjusted model additionally adjusted for maternal age at delivery (by natural cubic splines) and paternal age at delivery (by natural cubic splines). The x-axis is age. The y-axis is the residuals of ASD around the black solid line, which represent the point estimate of spontaneous relative risk (RR) of ASD. The fitted line (solid blue line with 95% confidence interval in light blue shadow) always overlapped the line of RR estimates (within the 95% confidence interval) throughout the time, which supported the proportional hazard assumption.

Supplementary Figure S4: Age-specific cumulative probability of Rheumatoid Arthritis for individuals with and without ASD, for the subpopulation 1985 – 1999

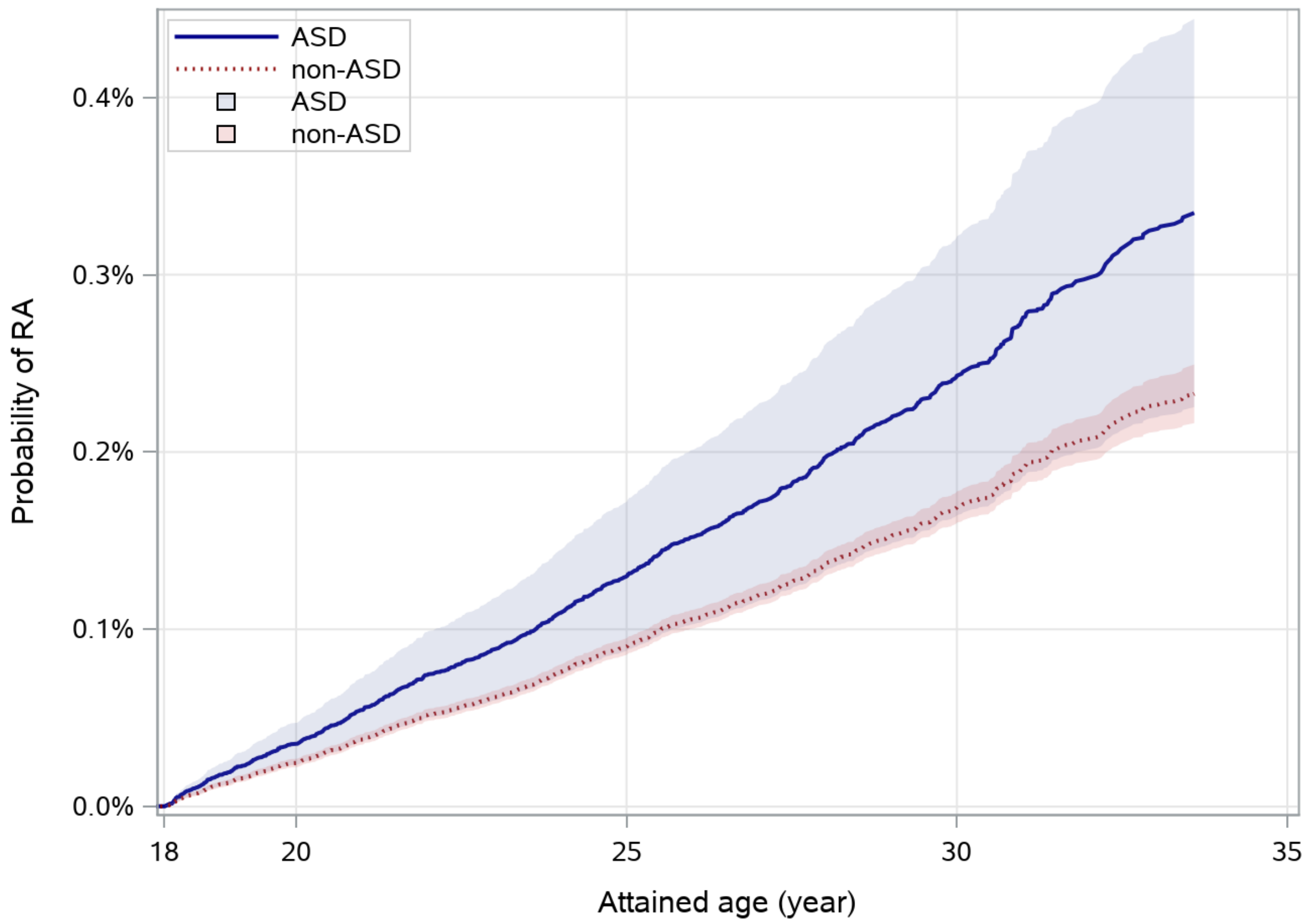

ASD: Autism Spectrum Disorders; RA: Rheumatoid Arthritis;

Footnote. Two curves: one for ASD (red solid line with red band), the other for non-ASD (blue dash line with blue bond). The bonds represented 95% confidence interval. The plots were based on a Cox model with these covariates: birth year by natural cubic splines (degree of freedom = 3) and sex. The x-axis represents age attained. The y-axis represents the cumulative probability of RA.

Supplementary Figure S5: Age-specific cumulative probability of RA for males and females, with and without ASD

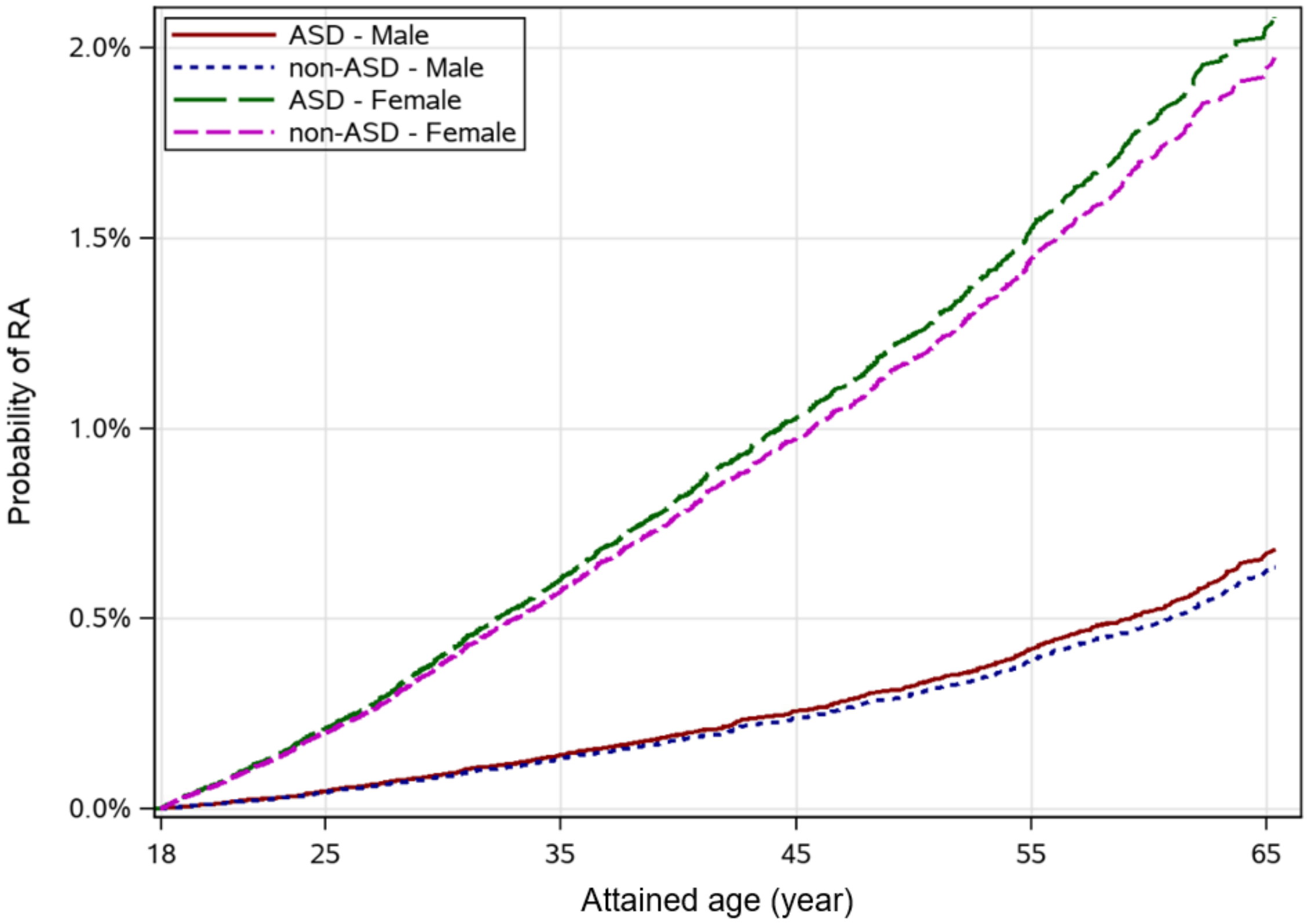

Footnote. Four curves: one for males with ASD (red solid line), one for females with ASD (green long dash line), one for males without ASD (blue short dash line), and one for females without ASD (purple medium dash line). The plots were generated by the Cox function. The x-axis represents age. The y-axis represents the cumulative probability of RA.

Supplementary Figure S6: The inspection of AFT Cox-Shell residuals of log-logistic distribution

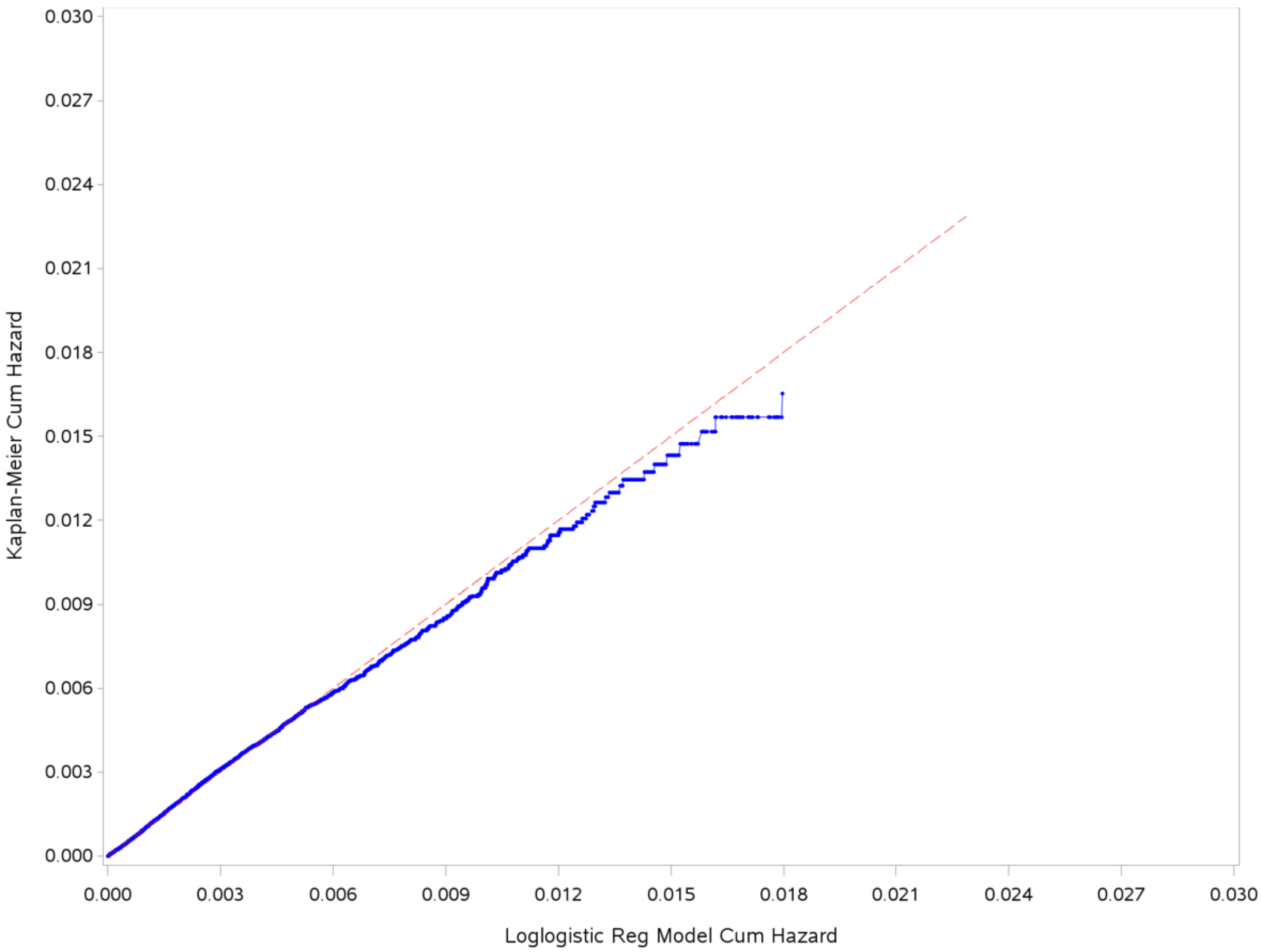

Footnote. Cox-Shell residuals[1] and examines whether the log-logistic distribution of survival times adequately fit the observed data. The x-axis is the cumulative hazard of the log-logistic distribution, which obtained the smallest AIC among the distribution assumptions in **Supplementary Table S5**. The y-axis is the Kaplan-Meier Cumulative Hazard. The fitted model is correct if the Cox-Snell residual has a log-logistic distribution, i.e., if the fitted curve (blue) is an approximately straight line through the origin and with a slope of 1 (reference line in red). Therefore, it supports the choice of log-logistic distribution.

Supplementary data S1: Interaction terms: (1) between birth year and ASD; (2) between sex and ASD

(1) between birth year and ASD

To assess if there are birth year specific (~ "age" in 1987) differences in ASD~RA association for individuals. We fitted two nested models for the examination. Model A adjusted for the birth year with natural cubic splines and sex. Model B additionally adjusted the interaction term of ASD and birth year. The natural spline cubic option automatically divides the birth year into 3 splines (df = 2) and accordingly Model B has 3 splines for the interaction term and 3 splines for the main effect of the birth year.

By assessing the goodness of fitting, Model A had a better performance with the AIC (147,589.6) slightly lower than the AIC of Model B (147,590.2). The main effect of birth year was statistically significant (P-value: <.0001) in both models but the interaction term of ASD and birth year was non-significant (P-value: 0.17).

We have no evidence that there are birth year specific (~ "age" in 1987) differences in ASD~RA association for individuals.

(2) between sex and ASD

To assess if there are sex-specific differences in ASD~RA association for individuals. We fitted two nested models for the examination. Model A was the same in (1). Accordingly a Model C additionally adjusted for ASD-sex interaction.

By assessing the goodness of fitting, Model A had a better performance with the AIC (147,589.6) slightly lower than the AIC of Model C (147591.6). The main effect of sex was statistically significant (P-value: <.0001) in both models but the interaction term of ASD and birth year was non-significant (P-value: 0.96).

We have no evidence that there are sex specific differences in ASD~RA association for individuals.

eReference*s*

1. Organization WH. World Health Statistics 2016 [OP]: Monitoring Health for the Sustainable Development Goals (SDGs). World Health Organization; 2016.
2. Control CfD, Prevention, Control CfD, Prevention. International classification of diseases, ninth revision, clinical modification (ICD-9-CM). In:2013.
3. Raqab MZ, Al-Awadhi SA, Kundu DJCiS-S, Computation. Discriminating among Weibull, log-normal, and log-logistic distributions. 2018;47(5):1397-1419.
4. Collett D. Modelling survival data in medical research. CRC press; 2015.
